# Challenges of Effective Clinical Teaching Organization at Public Medical Universities in Poland: A Delphi Method Study

**DOI:** 10.3390/medsci14050594

**Published:** 2026-09-21

**Authors:** Pawel Zawieja, Anna Lipert, Remigiusz Kozlowski

**Affiliations:** 1 Department of Management and Logistics in Health Care, Medical University of Lodz, Narutowicza 60, 90-136 Lodz, Poland; 2 Cardiovascular and Metabolic Diseases Prevention Laboratory, Department of Preventive Medicine, Medical University of Lodz, Zeligowskiego 7/9, 90-752 Lodz, Poland; anna.lipert@umed.lodz.pl

**Keywords:** clinical learning environment, hospital-based teaching, medical students, workplace learning

## Abstract

**Background/Objectives**: Clinical teaching as a form of interpersonal education organized in a real clinical setting is very dynamic and sensitive to many factors that may influence its effectiveness. To strengthen clinical education organization, responsible bodies should meet emerging challenges, which are best understood from the perspective of the direct recipients and providers of clinical teaching. Therefore, the aim of this study was to explore and analyze the existing challenges of clinical teaching organization in public medical universities in Poland from students’ and academics’ perspectives. **Methods**: A modified two-round Delphi study was conducted in six Polish medical universities. Round 1 included 218 experts (131 students and 87 academics), while 124 experts completed Round 2 (attrition rate: 43.1%). Consensus was predefined as a median score ≥ 4.0 and IQR ≤ 1.0. **Results**: Items with the highest mean values (approximately 4.4 on a five-point Likert scale) indicated a clear perception of space shortages and demand for updated teaching methods. The exploratory correlation revealed two broad thematic patterns: (1) space and overcrowding; (2) teaching methods. **Conclusions**: The findings suggest that stakeholders perceive educational space, overcrowding and modernization of teaching methods as important areas for improvement in clinical teaching.

## 1. Introduction

Clinical teaching is a form of interpersonal education between a clinician and a student in a clinical setting that is focused on patients and their real problems and in which the patient is also directly involved in the process [1]. During medical studies, there should be as much clinical teaching exposure as possible to develop several skills, i.e., history taking, physical examination, clinical reasoning or decision making [1]. Also, bedside and out-patient clinic tutorials orientate students to the cultural and social aspects of the clinical environment and, thus, shape their professional values [2]. However, organization of effective clinical teaching is much harder than it seems, and it is difficult to find one best approach common to all universities due to the great variability in the organization of medical education systems/approaches/models in Europe.

There are several different medical education curricula in Europe, which depend on regional, political and other contextual factors [3]. Also, the implementation of the principles of the Bologna system across Europe is highly variable [4]. For example, Belgium declares its higher education provision to be ‘Bologna-compliant’. In other countries, including Poland, major exceptions include professional degree qualifications, such as medicine or dentistry [4], which still require full 5 years of study when the entire curriculum is completed. In Poland, there is also a sixth year of study, which is a year of practice previously called a medical internship after studies [5]. The first and second years of study include theoretical subjects, such as anatomy, biology, biophysics, chemistry, etc. From the third year, clinical teaching in small student groups begins and is held in hospitals with patients. The curriculum also includes one-month internships in hospital wards held after each year during the summer holidays. In Poland, the whole system of medical studies is regulated by the Act on the Law on Higher Education and the Act on the Professions of Physicians and Dentists [6,7].

Teaching in the clinical environment is demanding and complex, requiring the clinical teacher to play many roles simultaneously, switching from one role to another at the same time and being more than only a medical expert [8,9]. Unfortunately, the large majority of clinical teachers around the world receive rigorous training in medical knowledge and skills but little to none in clinical teaching [10]. Also, adequate preparation for clinical classes is made more difficult by the fact that physicians are busy in their own clinical practice. Being effective becomes more challenging in the context of expanding clinical responsibilities and shrinking time for clinical teaching [10]. Research shows that students’ classes in hospital wards are usually incidental, not planned, and unorganized [11]. There is evidence that the effectiveness of clinical teaching and students’ perceptions of the clinical learning environment are influenced by the quality of students’ relationships with the clinical faculty [12]. Factors such as a shortage of healthcare staff, a lack of clinical instructors and high patient loads for staff in the ward may contribute to the formation of an unfavorable clinical learning environment. To mitigate these factors, various innovative approaches have also been introduced, such as simulated ward rounds [13], simulation-based learning experiences (SBLEs) [14], and AI-informed practice grounded in outcome-based education [15].

Due to the fact that the clinical environment is very dynamic and sensitive to many factors that may influence effective clinical teaching, it needs serious attention from responsible bodies to strengthen clinical education organization. While the challenges of clinical education are widely debated, the literature remains fragmented across disciplines [16]. Research in medical education predominantly focuses on teaching competencies and methods [14,15,17,18,19,20]. In contrast, broader structural constraints, such as facility shortages or overcrowded wards, are more extensively documented in the nursing, midwifery, and veterinary fields. A significant research gap persists regarding how these organizational barriers impact undergraduate medical students and academic physicians within curricula like Poland’s. Lacking a comprehensive global systematic review, this study narrows its focus to identify these parallel structural challenges within public medical universities. The aim of this study was to identify and explore the spatial, organizational, and educational challenges affecting clinical teaching from the perspectives of students and clinical teachers, and to determine potential approaches for improving the quality and effectiveness of clinical education in healthcare settings.

## 2. Materials and Methods

### 2.1. Study Design and Procedure

**Figure 1:**
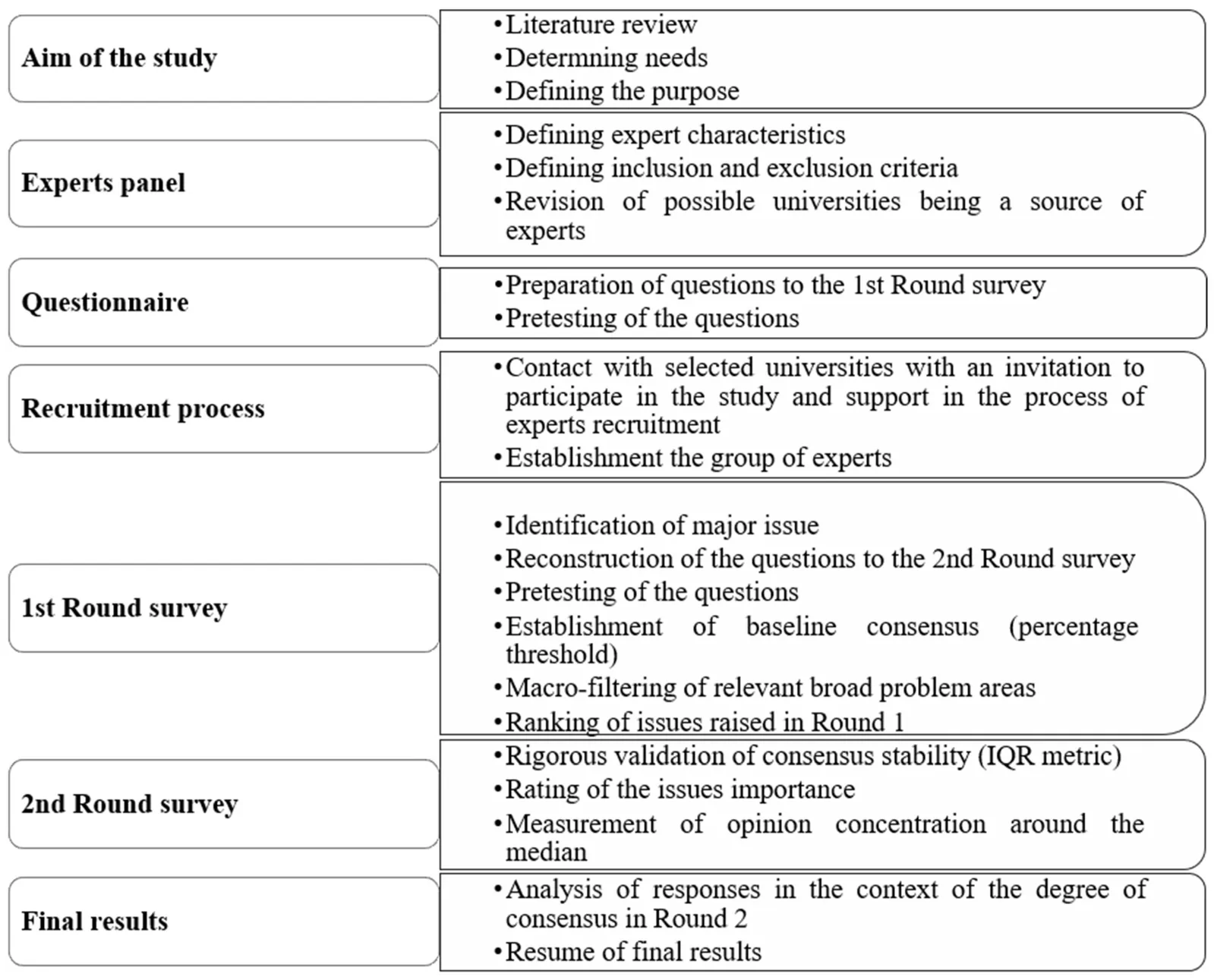
Flowchart of the research process.

**Figure 2:**
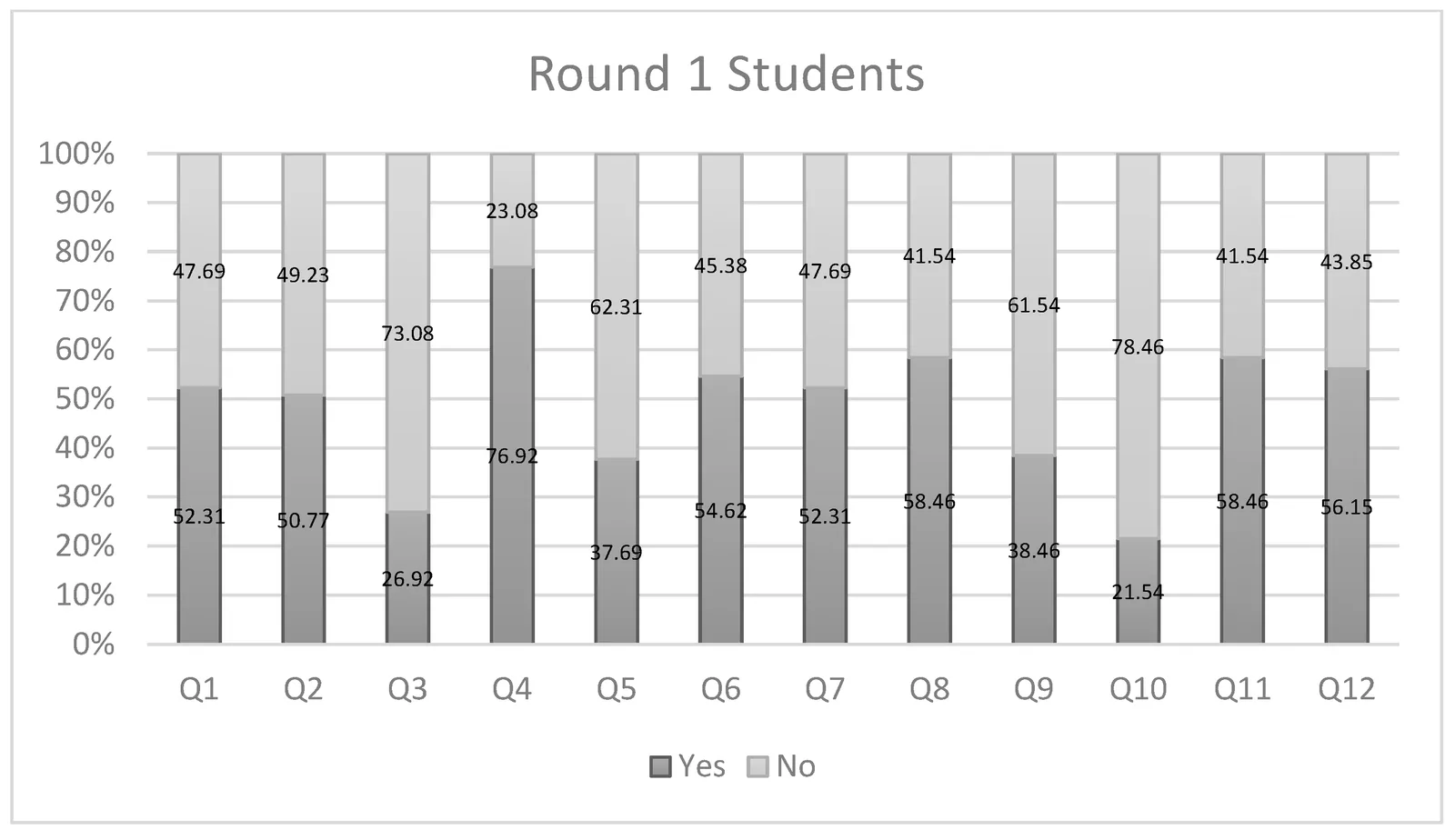
Distribution of student responses and initial consensus patterns across items in Round 1.

**Figure 3:**
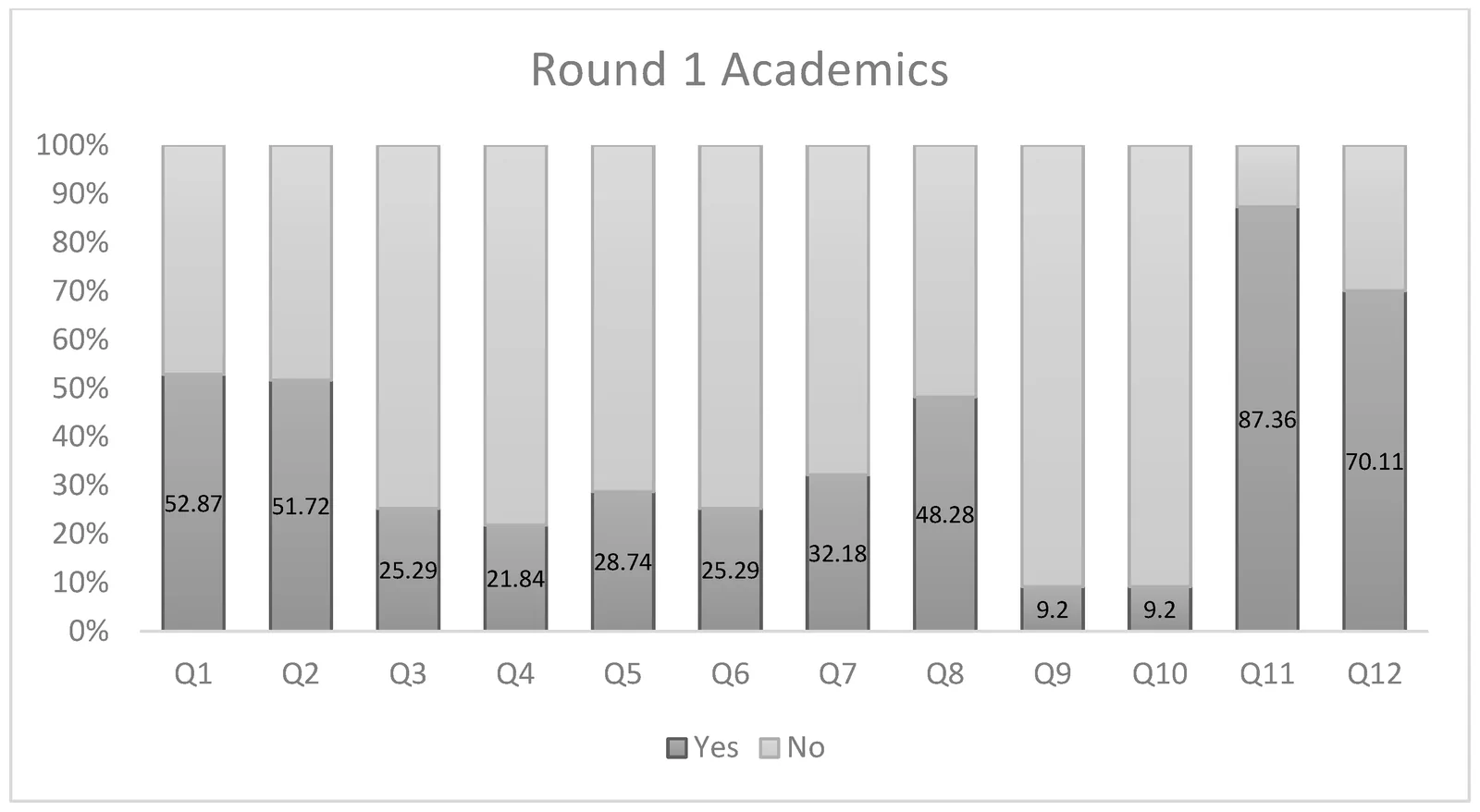
Distribution of academics’ responses and initial consensus patterns across items in Round 1.

This was a descriptive multicenter study conducted using the modified Delphi method, with two iterative rounds specifically designed to optimize expert engagement and mitigate respondent fatigue. The Delphi method uses a series of questionnaires to collect data from the panel of experts [21,22]. Each iteration of questionnaires represents feedback obtained from the panel of experts, allowing them to reassess their opinions and judgments on a specific topic. Figure 1 outlines the process. In Round 1, detailed descriptive statements regarding the problem areas were presented to the panel. The purpose of the initial iteration was to identify (a) the current challenges of clinical teaching organization related to clinicians’ availability and (b) the perceived needs and priorities for improving teaching and learning opportunities in clinical settings. To minimize the cognitive burden and reduce the time required from the participants, a dichotomous (yes/no) agreement scale was utilized. Crucially, an open-ended option was included to preserve the exploratory nature of the Delphi method, allowing experts to contribute additional insights. The primary objective of Round 1 was exploratory rather than conclusive. It was not intended to establish consensus but to explore the distribution of opinions and identify areas requiring further refinement across both subgroups (Figure 2 and Figure 3). The observed patterns of agreement and disagreement, together with the qualitative comments, informed the development of the Round 2 statements. Representative themes derived from qualitative comments collected in Round 1 are presented in Table 1. Open-ended comments collected in Round 1 were reviewed by the research team. Similar comments were grouped into broader problem areas. The response patterns and qualitative inputs from Round 1 served as the conceptual foundation for extracting, expanding and categorizing the core problem areas into more detailed statements. In Round 2, these newly synthesized areas were returned to the experts using a 5-point Likert scale to measure their perceived importance and determine the final group consensus. Consequently, consensus was formally evaluated only in Round 2.

Given the modified Delphi design and the recognized risk of expert fatigue, participant attrition, and potential response bias in subsequent rounds, this study was intentionally concluded after Round 2. The interquartile range (IQR) was employed as the primary consensus metric, utilizing a standard threshold of IQR ≤ 1.0 [23,24]. The data (Table 2) demonstrated that a satisfactory level of consensus was successfully established across most items. As this study employed a modified two-round Delphi design, Round 2 constituted the formal consensus assessment stage. The predefined consensus criterion was achieved for most items, indicating a satisfactory level of internal consensus within the final evaluation round and justifying the conclusion of the Delphi process after Round 2.

Analysis of Round 1 responses identified two major thematic domains: (A) organizational aspects of clinical teaching and (B) teaching, learning and assessment in clinical education. The present article focuses on Domain A; therefore, only findings related to this domain are reported here.

This study was pseudonymized. At the end of Round 1, participants were asked to voluntarily provide an e-mail address if they agreed to be contacted for the subsequent Delphi round. Provision of an e-mail address was considered consent to receive study questionnaires during the next Round. Contact information was used solely for the purpose of distributing the Round 2 questionnaire and was not linked to survey responses during data analysis. Therefore, the anonymity of responses was maintained throughout the analytical phase of this study. As a non-interventional educational study that did not involve patients, medical procedures, biological material, or clinical data, formal bioethics committee review was not required under the applicable institutional framework.

To develop the first-round questionnaire, three publications [2,24,25] were selected because they synthesized research on medical education published during the 10 years preceding this study. Recurring issues and themes identified in these sources were used to formulate the initial questionnaire items. In addition, data from previously conducted surveys on the quality of education at the university were used, which also served as an inspiration to conduct the presented study. These surveys primarily addressed student satisfaction with their learning conditions, and the recurring themes identified in these reports were used to refine and supplement the initial questionnaire items. Before the official launch of Round 1, the pre-test of the questions was piloted among 10 individuals representing both target stakeholder groups, including six medical students and four academic teachers who were not included in the main analysis. The goal of this stage was only to assess the clarity, readability and unambiguity of the statements, ensuring the validity of the research instrument and preventing potential interpretation errors during the main phase of this study. The pilot testing indicated the good comprehensibility and relevance of the questionnaire items. Consequently, only minor editorial modifications and wording refinements were required. A few participants also suggested expanding selected topics by adding a small number of questions. These suggestions were incorporated into the final version. In the Round 2 questionnaire, the experts were asked to rate from 1 to 5 on a Likert scale the importance of each of the issues diagnosed in Round 1. A 5-point Likert-type scale, where 1 indicated ‘very unimportant to address’, 2 indicated ‘unimportant to address’, 3 indicated ‘no opinion’, 4 indicated ‘important to address’, and 5 indicated ‘very important to address’, was used in the Round 2 questionnaire.

In each round, the questionnaires were posted via e-mail with a link to access the online form. At the beginning of each round, a questionnaire included information explaining the aim of this study and the main principles of this technique. Also, the participants were informed that the survey was anonymous and that completing the questionnaire was equal to agreeing to participate in this study. The data collection lasted for a period of 12 months.

**Figure 4:**
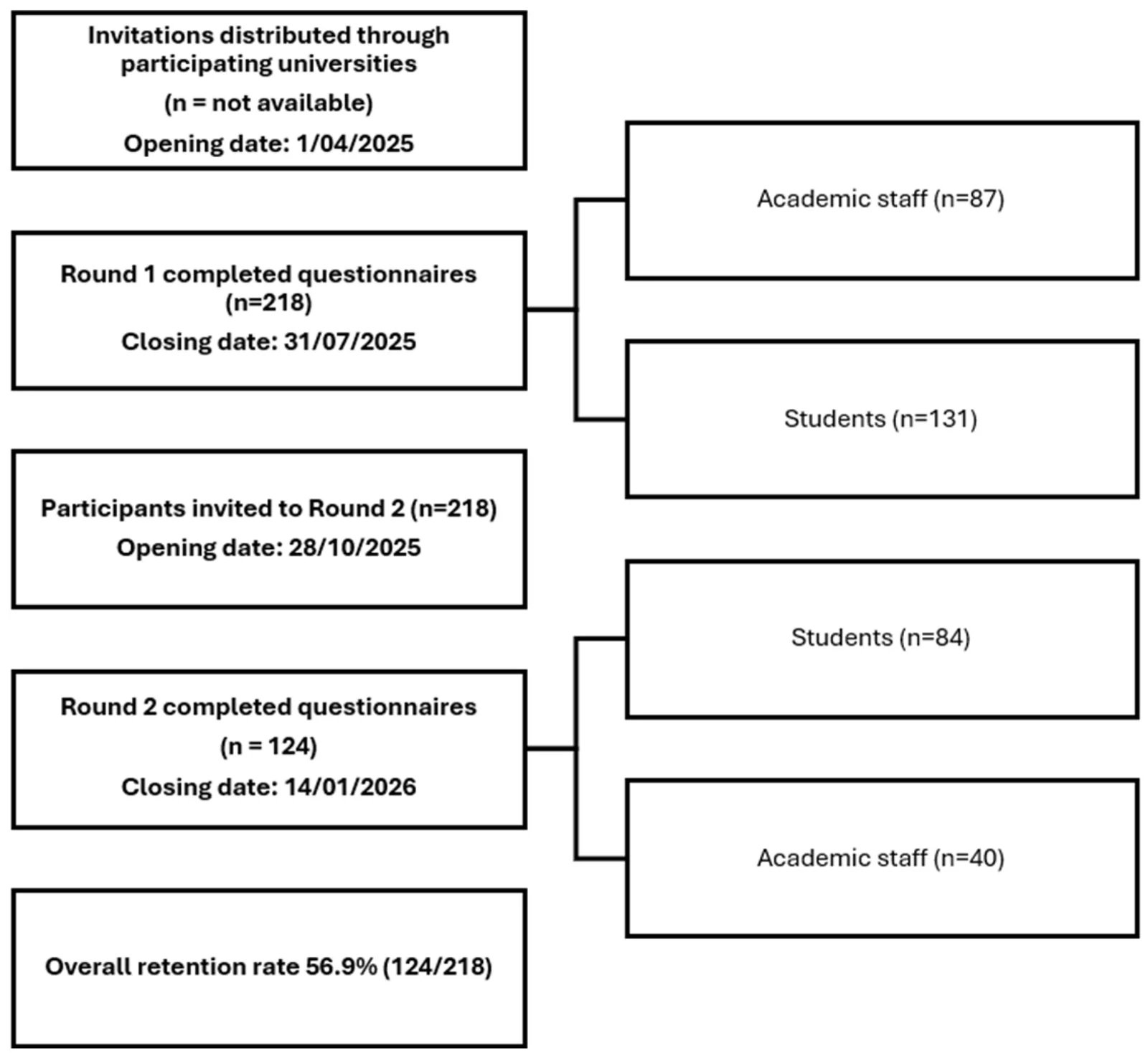
Flow of participants throughout the two-round Delphi process. Recruitment was conducted through participating universities. Owing to the anonymous nature of participation, the number of invitations distributed and institution-specific participation rates could not be determined. The diagram presents the number of completed questionnaires in each Delphi round and participant retention between rounds.

The final sample after Round 2 consisted of the answers provided by 84 students and 40 academic staff. An overall sample retention rate of 56.9% between the 1st and 2nd Rounds indicated a total sample attrition rate of 43.1%. Group-specific engagement varied significantly: students demonstrated higher persistence, with a 64.1% retention rate, whereas academic teachers showed a notable decline in participation, resulting in a lower retention rate of 46.0% (Figure 4).

### 2.2. Participants

**Figure 5:**
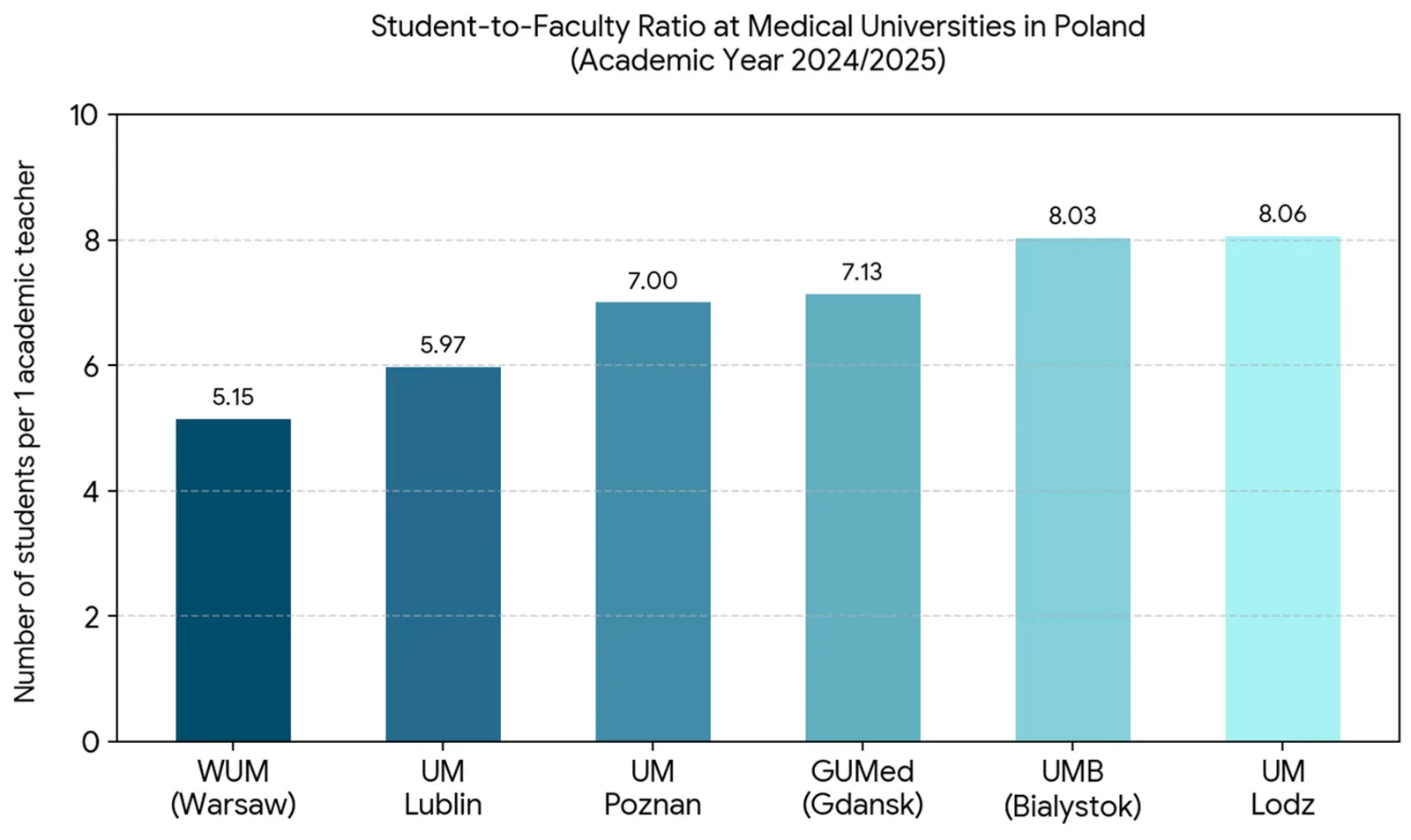
Institutional characteristics of participating medical universities based on publicly available data (2024/2025 academic year).

The selection of universities was purposeful and took into account regional diversity and the importance of selected academic centers in educating medical personnel in Poland. In general, there are nine Public Medical Universities in Poland (Łódź, Warsaw, Lublin, Silesia, Białystok, Wrocław, Poznań, Gdańsk, and Szczecin) and two medical colleges (large faculties with greater autonomy following the merger of universities) at the Jagiellonian University and Toruń University. However, 6 out of 9 universities agreed to participate in this study. Figure 5 presents the publicly available institutional characteristics of the participating universities and is provided solely to offer contextual information regarding the educational environments in which this study was conducted. Due to the fact that the recruitment process was anonymous and institution-specific, participation data were not collected, and the figure should not be interpreted as reflecting participant recruitment or participation rates. The participating universities operate in large academic and urban centers with significant roles in the healthcare system and medical education in Poland.

In this study, an attempt was made to select a multicenter sample of academics and students to obtain a panel of experts who personally experience the process of clinical classes and, therefore, within the terms of this study, represent a panel of ‘informed experts’ by reason of their day-to-day involvement in this process. An expert in the Delphi method is defined as someone with knowledge and experience on a particular subject matter [26]. The predefined criteria were as follows: (1) being an academic or student at a medical university; (2) involvement in clinical teaching/learning. The invitations were distributed through the authorities of participating medical universities. The research team did not directly select potential participants and had no control over which eligible individuals ultimately received the survey invitation. However, the recruitment process was designed to maximize representation of 2 clinical education stakeholder groups—academic teachers and medical students—people who provide a distinct and valuable lens for identifying the most important challenges that need to be improved for clinical teaching success. Student participants were eligible to be included in this study if they were enrolled in a medical program and actively participated in clinical classes. Academic participants were eligible to be included in this study if they were employed academic staff involved in delivering clinical teaching at public medical universities. In the Polish medical curriculum, clinical teaching begins from the third year of study. Therefore, all eligible students were expected to have direct exposure to clinical teaching. Also, academic participants were eligible if they were employed academic staff actively involved in delivering clinical teaching at public medical universities. Detailed information on year of study, academic rank or teaching experience was not collected in order to preserve participant anonymity and encourage candid responses.

### 2.3. Statistical Analysis

All the data were analyzed using the TIBCO Statistica software, version 13.3 (TIBCO Software Inc., distributed by StatSoft Polska Sp. z o.o.).

A group mean score was calculated for each of the 13 statements evaluated in Round 2. These statements were derived from and expanded upon the thematic areas identified in Round 1. The median and interquartile range were operationally calculated for each statement to determine whether formal Delphi consensus was achieved. The mean response scores and standard deviations were utilized strictly as descriptive statistics for exploratory purposes, specifically to summarize the perceived overall importance of each challenge and to facilitate ranking and comparison across items, rather than for formal statistical inference. To examine the relationships between the analyzed variables—diagnosed challenges—Spearman’s rank correlation coefficient was utilized. Spearman’s correlation assesses the strength and direction of the monotonic relationship between variables without assuming a linear relationship or normal distribution of the data. There were no missing data in the Round 2 dataset due to the mandatory response format of the electronic questionnaire. Therefore, the full sample sizes were preserved for all statistical calculations. Statistical significance was set at *p* < 0.05.

## 3. Results

### 3.1. Descriptive Comparison Between Students and Lecturers

**Figure 6:**
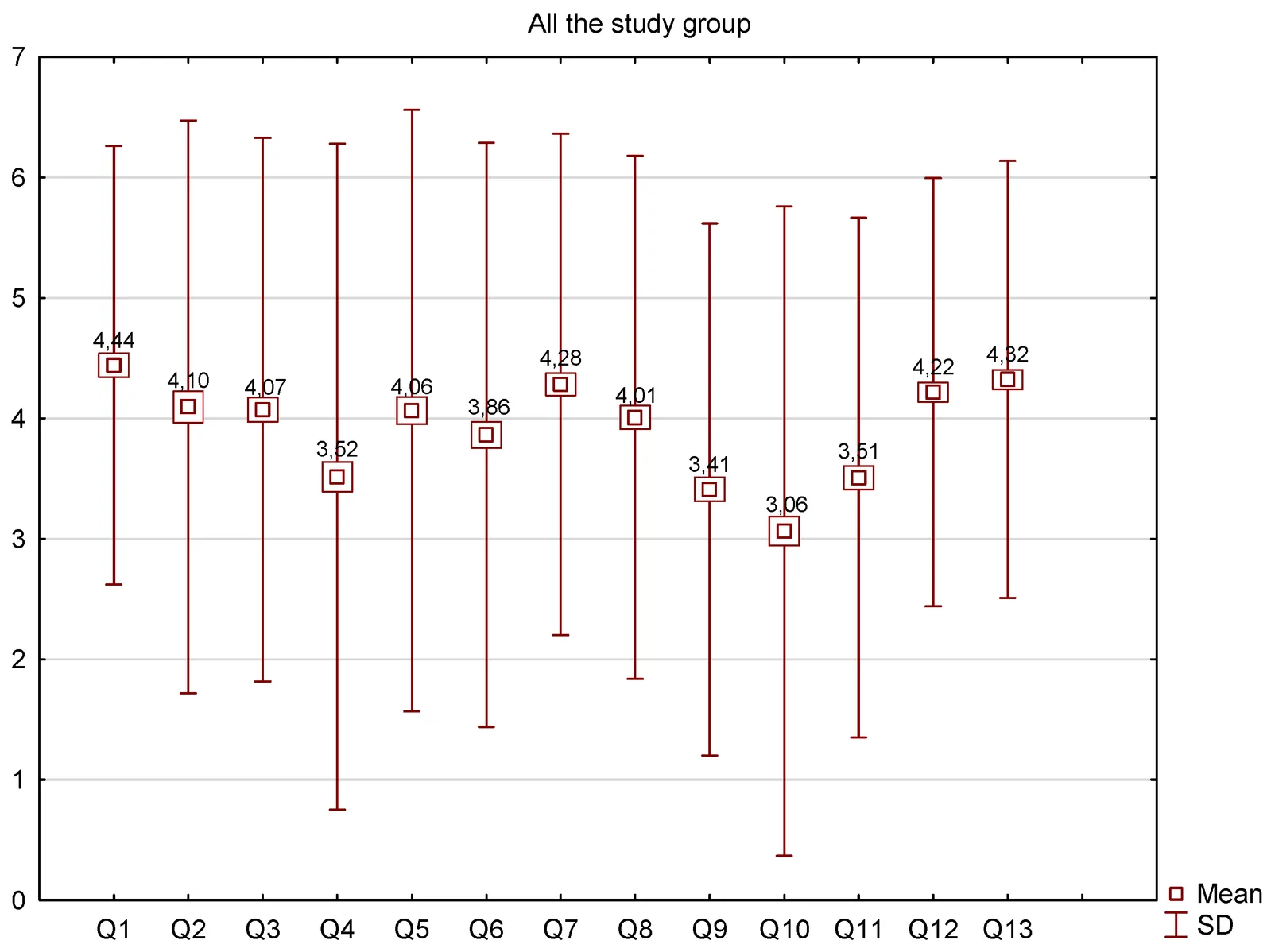
Mean expert ratings regarding the existence of selected problems in the overall study group. Q1: No cabinets; Q2: No place for a break; Q3: Lack of teaching rooms; Q4: Too small teaching rooms; Q5: Too numerous student groups; Q6: Classes with the patient in a separate place; Q7: Too small rooms in clinics; Q8: Too many students to work with patients; Q9: Too few teaching activities; Q10: Lack of computer rooms; Q11: More self-learning methods; Q12: More Cased-Based Learning[EE1.1][RK1.2]; Q13: More simulated classes.

**Figure 7:**
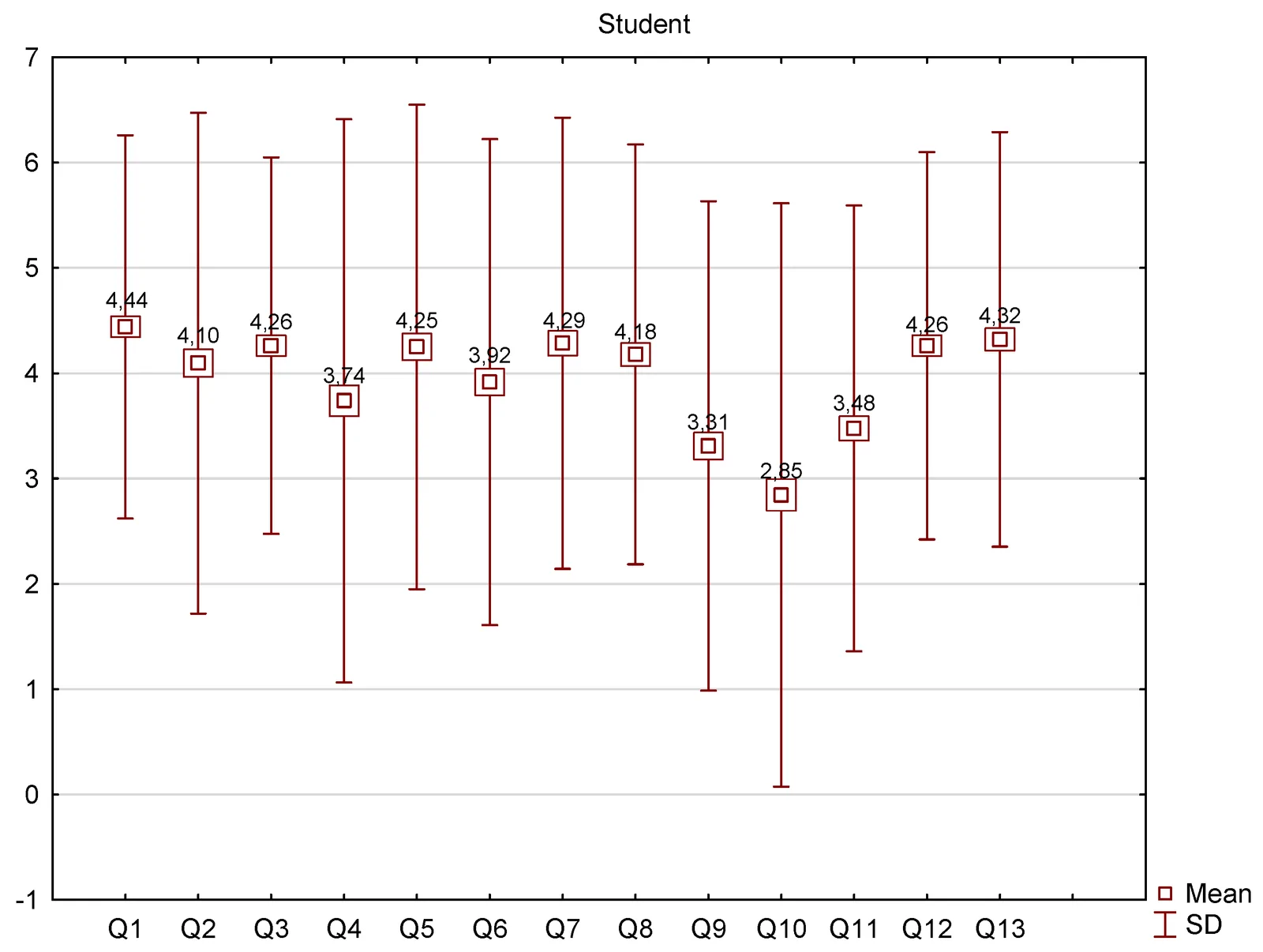
Mean expert ratings regarding the existence of selected problems among students. Q1: No cabinets; Q2: No place for a break; Q3: Lack of teaching rooms; Q4: Too small teaching rooms; Q5: Too numerous student groups; Q6: Classes with the patient in a separate place; Q7: Too small rooms in clinics; Q8: Too many students to work with patients; Q9: Too few teaching activities; Q10: Lack of computer rooms; Q11: More self-learning methods; Q12: More Cased-Based Learning[EE1.1][RK1.2]; Q13: More simulated classes.

**Figure 8:**
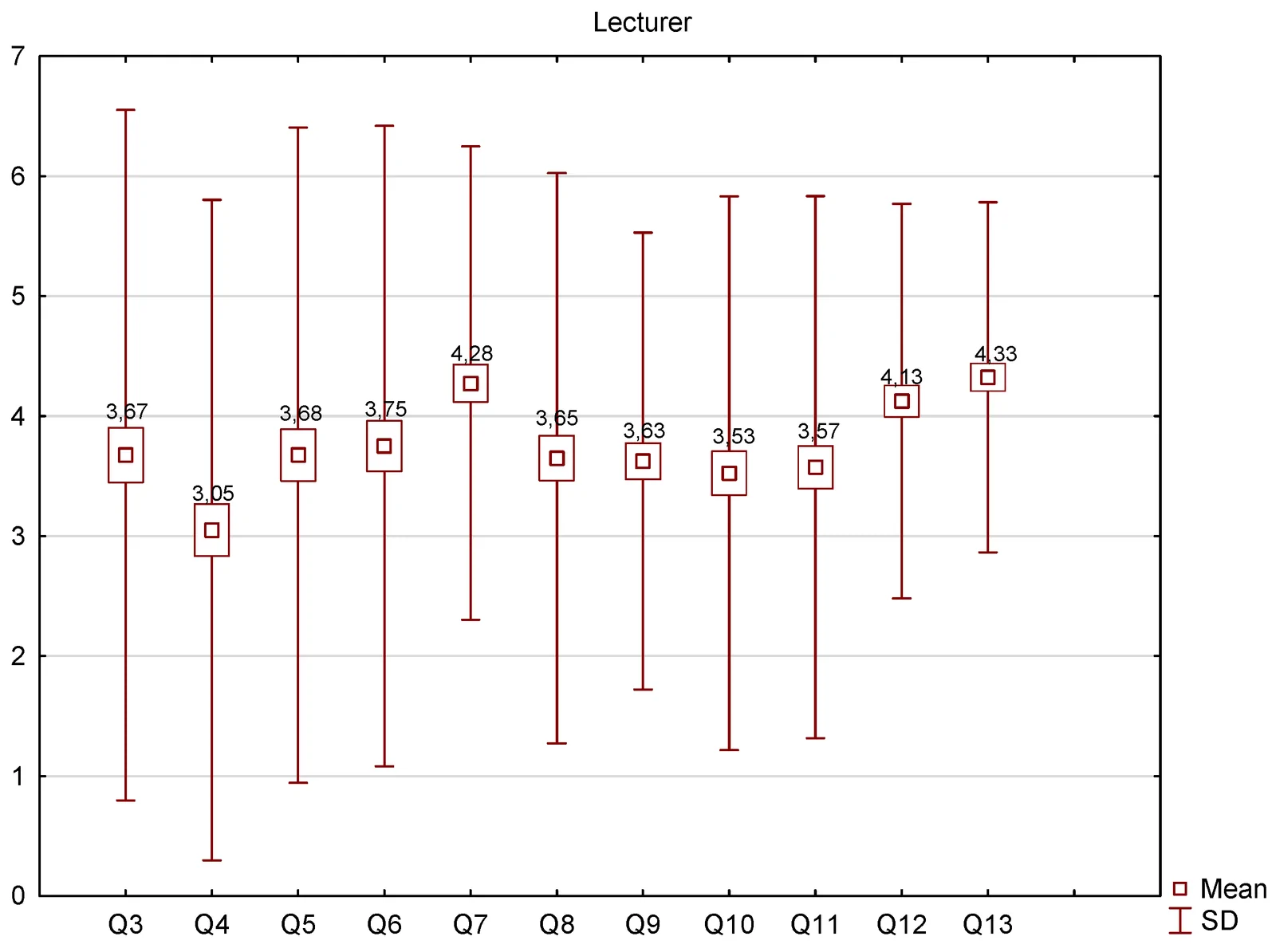
Mean expert ratings regarding the existence of selected problems among lecturers. Q3: Lack of teaching rooms; Q4: Too small teaching rooms; Q5: Too numerous student groups; Q6: Classes with the patient in a separate place; Q7: Too small rooms in clinics; Q8: Too many students to work with patients; Q9: Too few teaching activities; Q10: Lack of computer rooms; Q11: More self-learning methods; Q12: More Cased-Based Learning[EE1.1][RK1.2]; Q13: More simulated classes.

Items with the highest mean values, approximately 4.4, indicated a clear perception of space shortages and demand for updated teaching methods. The lowest-rated items still represented perceived issues, but they ranked lower relative to other items. Nearly all items showed large SD values, some extending across 3–4 points. This indicates that respondent opinions varied widely; there was not much agreement within the full group; and different subgroups (students vs. lecturers) likely perceived problems very differently. This aligns with the earlier table data showing students were more critical than lecturers (Figure 6). Students reported high means (similar to but slightly higher than those of the overall group). This suggests that students may have perceived environmental factors as more important (space and room size) and also that students desired innovation in teaching more (simulation and self-directed learning). Students showed large SDs as well, although some items were slightly more consistent than in the full group. This means that student views were diverse but showed clearer patterns than the mixed sample. Students appeared to show a higher level of agreement on space/room problems and demand for modern teaching methods (Figure 7). Academic teachers reported slightly lower means across most categories, confirming that academic staff may have perceived these environmental and structural challenges as less challenging than the students. Despite this, academics still expressed more desire for educational innovation, giving their highest scores to the demand for more simulated classes and more Case-Based Learning. Furthermore, the large SDs revealed that academic teachers’ opinions were highly diverse and lacked a unified agreement, indicating heavily fragmented viewpoints within the teaching staff (Figure 8).

In general, students reported higher mean scores for the identified challenges, which may suggest they perceived clinical environment problems more strongly. The overall SDs for the complete group were inflated because lecturers tended to give lower scores, increasing variability. The most prominent issues in both groups were lack of space (cabinets and clinical rooms), overcrowding (too many students) and the need for modern teaching methods (simulation, CBL, and self-learning).

### 3.2. Overall Correlation Matrix

Spearman’s rank correlation coefficients were calculated as an exploratory analysis to investigate the relationships between the ratings of individual challenges. Most correlations were moderate and positive, indicating that people who perceived one organizational problem (e.g., lack of teaching rooms) tended to perceive others as well (e.g., too many students in clinics). The strongest positive correlations (r ≥ 0.45) were observed between (a) No place for a break vs. Classes with the patient in a separate place (r = 0.51); (b) Too small teaching rooms vs. Too small rooms in clinics (r = 0.45); (c) Too small rooms in clinics vs. No place for a break (r = 0.46); (d) More case-based learning vs. More simulated classes (r = 0.36); (e) Too numerous student groups vs. Too small rooms in clinics (r = 0.40); and (f) Too many students to work with patients vs. Too small teaching rooms (r = 0.33). The exploratory correlation analysis revealed two broad patterns of related items. The first pattern of correlated items was space and overcrowding, so items related to too many students, small rooms, lack of teaching rooms, and lack of break space were all linked. This means respondents perceived these problems as interconnected, likely reflecting a structural space shortage. The second pattern of correlated items was teaching with different methods, so items such as more self-learning methods, more CBL, and more simulation correlated with each other. The overall correlations were consistent and made theoretical sense (Table 3).

Compared with the general matrix, students demonstrated higher coefficients, indicating a more consistent pattern of perceived structural problems. Students consistently linked overcrowding to room size problems, suggesting these issues strongly shaped their learning experience. The teaching methods pattern was also present but slightly weaker than that of organizational issues (Table 4).

Lecturers formed a very consistent thematic group of teaching methodology improvements, similar to students but even greater. Lecturers also showed strong correlations within the overcrowding/space pattern. Lecturers, like students, perceived space and student overload as interconnected problems. But unlike students, their strongest pattern concerned teaching methods, suggesting educators were highly aware of the need for modern teaching improvements (Table 5).

## 4. Discussion

Clinical teaching is based on structured classes in the form of ward rounds, seminars or case presentations, but it can also be completed by unplanned opportunities that present themselves during the routine medical services delivered by the hospital. Students have the opportunity to participate in real-life situations, observe rare cases and benefit from the contributions of a variety of hospital staff with various perspectives and backgrounds [27]. For this reason, clinical teaching is considered to be one of the most valuable forms of education in the curriculum of future physical practitioners. However, due to the fact that clinical teaching takes place in the natural conditions of the hospital, clinical teachers have to fulfil two main roles in the workplace: teaching students and providing clinical care for patients [8]. Unfortunately, research shows that clinical teaching is perceived as demanding and difficult to balance when the provision of clinical care outweighs teaching [16,28]. The present study demonstrates descriptive differences between students’ and lecturers’ mean ratings regarding organizational challenges in clinical teaching. Students generally reported higher mean values across nearly all items, particularly regarding lack of space, overcrowded groups and insufficient clinical room availability. The same result was observed in Hakim’s study, in which the opinion of most of the students revealed that there was a lack of educational facilities and other amenities for both students and educators [26]. Moreover, students experienced difficulties in cooperation between medical staff members and students in education centers, which caused problems in clinical education [25]. Some studies have raised even the issue of violence directed towards students by medical staff members or patients, although these results are from studies among nursing students [29]. Other barriers mentioned in the literature include a large number of students in groups in comparison with a smaller number of patients on the ward or insufficient physical space and educational classrooms [30,31]. In contrast, lecturers reported lower descriptive mean scores for the same problems, which may reflect their adaptation to existing institutional conditions and broader understanding of systemic constraints. In our study, these observations should be interpreted as descriptive patterns rather than statistically verified differences between groups. However, in Hakim’s study, the mean of students’ ideas was also higher than the mean of educators’ ideas, and the differences were statistically significant in all sections [26]. This may suggest that students experience structural limitations more directly during everyday clinical exposure.

Although high standard deviation values indicated considerable variability in opinions across respondents, certain patterns of correlated items and shared areas nevertheless emerged, revealing two strong and coherent groups of structural challenges related to space limitations and overcrowding and modernization of teaching methods. While analyzing the pattern of correlated items related to space limitations and overcrowding, it was observed that significant positive correlations existed between insufficient teaching rooms, small clinical spaces, a lack of break areas and excessive student numbers. When asked about the highly ranked attributes of physical learning spaces, medical students indicated access to rooms, such as clinical ward discussion rooms, external consultation units, auditoriums, classrooms or hospitalization rooms, while also indicating silence and the seating furniture [32]. This suggests that students perceive these issues as interconnected elements of one broader infrastructural problem, which, in turn, translates into the effectiveness of clinical teaching. Evidence confirms that the attributes of physical spaces can impact experiences and learning engagement and facilitate or hinder learning in general [32]. Furthermore, overcrowded student groups, apart from making it difficult to access the patient, may also lead to ineffective teaching, increased workload and heightened stress for lecturers and reduce students’ participation and hinder their performance [33]. In relation to the second pattern of correlated items, which concerned the demand for modernization of teaching methods, both students and lecturers demonstrated consistent correlations within this methodological domain, with lecturers showing an even stronger internal consistency in this cluster. Both groups indicated a demand for greater use of simulation, case-based learning and self-directed approaches, which is in line with general transformation in higher education towards new student-centered active methods [34]. Although innovative forms of teaching mentioned here were highly valued by both groups, they should be viewed as complementary rather than a substitute for bedside teaching, which remains essential for learning. Therefore, the observed demand for simulation should be interpreted as support for a balanced integration of modern educational approaches rather than replacement of direct patient contact. Also, the evolution of digital technologies brings significant changes in higher education [35], which requires clinical settings and universities to demonstrate flexibility in digital method integration and adaptation for both student needs and educational requirements. However, in the present study, the coexistence of strong structural and methodological clusters suggests that the perceived need for innovative teaching methods may partly arise also as a response to infrastructural constraints. The results indicate that both groups concurrently highlighted the need for structural space expansion and pedagogical modernization. While it is possible that use of innovative digital or simulation methods could theoretically help alleviate some structural space pressures, further research is required to establish any direct relationship between these issues. In the present study, these findings are interpreted strictly as parallel areas that may require institutional attention.

This study is one of the few that explores current challenges in clinical teaching based on direct feedback from medical students and lecturers. While previous publications in this area have focused primarily on nursing or midwifery students [26,36], this study provides a new perspective and fills a significant gap in the literature. Due to the pioneering nature of the topic and the unique nature of the data collected, this study is exploratory in nature, allowing for the discovery of valuable lessons for the education of future physicians. In addition, data were collected using the Delphi method, which is one of the most reliable expert methods for forecasting and building consensus [37], especially when hard historical data are lacking, and has gained acceptance in diverse fields of medicine [25]. Its high credibility is based on participant anonymity and the elimination of pressure from authority figures.

Despite the rigorous Delphi methodology, this study has some limitations which should be emphasized. The first is the risk of an increased attrition rate between survey waves, resulting in a differential attrition rate observed between the two Delphi rounds. While medical students demonstrated a high persistence, with a 64.1% retention rate, academic teachers showed a more notable decline in participation, resulting in a lower retention rate of 46.0% in Round 2. However, in the case of the research group consisting of medical staff, who are burdened with numerous teaching responsibilities, the risk of premature withdrawal was high. A potential decline in participation in subsequent iterations could have affected the stability and representativeness of the final consensus. Furthermore, this imbalance in subgroup retention could potentially have altered the final composition of the expert panel and introduced response bias. Therefore, the observed descriptive differences between the students and academics must be interpreted with caution, as they represent general descriptive patterns rather than statistically verified group differences. The second limitation relates to the expert recruitment procedure and the lack of full control over the final demographic and professional composition of the sample because detailed demographic and professional characteristics of participants were not collected. Therefore, the representativeness, diversity, and level of expertise of the participant panel could not be formally assessed. Although the study protocol precisely defined the inclusion criteria for “experts,” official requests for participation in this study were submitted centrally to the authorities of the partner universities, which then independently decided how to distribute the survey internally. This introduced the possibility of selection bias. Additionally, because institution-specific participation data were not collected, potential clustering effects at the university level could not be evaluated, and the generalizability of the findings should, therefore, be interpreted with caution. In addition, the questionnaire deliberately omitted detailed questions about the respondent’s academic degree, length of service or specific organizational unit to ensure maximum security and confidentiality for participants. The academic and medical environment can be highly hierarchical, and a critical assessment of clinical teaching conditions could raise concerns about identification and potential professional consequences, which could discourage potential experts from participating in the study or lead to insincere responses.

## 5. Conclusions

The findings of this study suggest that stakeholders perceive educational space, overcrowding and modernization of teaching methods as important areas of improvement in clinical teaching. These findings may help inform future institutional discussions and the development of interventions aimed at improving the clinical teaching environment. Further research is needed to evaluate the effectiveness of specific organizational and educational strategies.

## Figures and Tables

**Table 1:** Qualitative analysis of open-ended comments from Round 1.

Domain	Theme	Examples of Issues Raised in Open Comments
(A) Organizational aspects of clinical teaching	Teaching infrastructure	Insufficient seminar rooms, discussions conducted in corridors, and limited student facilities and changing areas
	Clinical learning environment	Excessive group sizes, limited access to patients, and overcrowded wards and clinics
	Teaching staff availability	Competing clinical duties, insufficient protected teaching time, and reduced accessibility of clinical teachers
	Organization and scheduling	Timetable gaps, inadequate advance planning, and difficulties related to location and scheduling of classes
(B) Teaching, learning and assessment in clinical education	Teaching methods	Need for greater use of case-based learning, problem-based learning and active participation
	Assessment	Concerns regarding examination methods; preference for clinical case-based assessment and practical examinations
	Feedback and competency development	Need for more constructive feedback and greater emphasis on practical clinical skills and communication competencies

**Table 2:** Consensus verification matrix of expert panel in Round 2.

Problem	All				Students				Academics			
	Me	IQR	Consensus Status	Interpretation	Me	IQR	Consensus Status	Interpretation	Me	IQR	Consensus Status	Interpretation
**Q1**	5.0	1.0	Consensus achieved	Good agreement, with most ratings clustered between 4 and 5.	5.0	1.0	Consensus achieved	Good agreement, with most ratings clustered between 4 and 5.	-	-	N/A	N/A
**Q2**	5.0	1.0	Consensus achieved	Good agreement, with most ratings clustered between 4 and 5.	5.0	1.0	Consensus achieved	Good agreement, with most ratings clustered between 4 and 5.	-	-	N/A	N/A
**Q3**	4.0	1.0	Consensus achieved	Good agreement, with most ratings clustered around 4.	4.0	1.0	Consensus achieved	Good agreement, with most ratings clustered around 4.	4.0	3.0	No consensus	Lack of consensus, with opinions highly dispersed.
**Q4**	4.0	3.0	No consensus	Lack of consensus, with opinions highly dispersed.	4.0	3.0	No consensus	Lack of consensus, with opinions highly dispersed.	3.0	2.0	No consensus	Lack of consensus, with opinions highly dispersed.
**Q5**	5.0	1.0	Consensus achieved	Good agreement, with most ratings clustered between 4 and 5.	5.0	1.0	Consensus achieved	Good agreement, with most ratings clustered between 4 and 5.	4.0	2.5	No consensus	Lack of consensus, with opinions highly dispersed.
**Q6**	4.0	2.0	Low agreement	Low consensus, with opinions moderately dispersed.	4.0	2.0	Low agreement	Low consensus, with opinions moderately dispersed.	4.0	3.0	No consensus	Lack of consensus, with opinions highly dispersed.
**Q7**	5.0	1.0	Consensus achieved	Good agreement, with most ratings clustered between 4 and 5.	5.0	1.0	Consensus achieved	Good agreement, with most ratings clustered between 4 and 5.	5.0	1.0	Consensus achieved	Good agreement, with most ratings clustered between 4 and 5.
**Q8**	4.0	1.0	Consensus achieved	Good agreement, with most ratings clustered around 4.	4.0	1.0	Consensus achieved	Good agreement, with most ratings clustered around 4.	4.0	1.5	High agreement.	Good agreement, with most ratings clustered around 4.
**Q9**	4.0	1.0	Consensus achieved	Good agreement, with most ratings clustered around 4.	3.5	2.0	No consensus	Lack of consensus, with opinions highly dispersed.	4.0	1.0	Consensus achieved	Good agreement, with most ratings clustered around 4.
**Q10**	3.0	2.0	No consensus	Lack of consensus, with opinions highly dispersed.	3.0	2.0	No consensus	Lack of consensus, with opinions highly dispersed.	3.5	1.5	No consensus	Lack of consensus, with opinions highly dispersed.
**Q11**	4.0	1.0	Consensus achieved	Good agreement, with most ratings clustered around 4.	4.0	1.0	Consensus achieved	Good agreement, with most ratings clustered around 4.	4.0	1.0	Consensus achieved	Good agreement, with most ratings clustered around 4.
**Q12**	4.0	1.0	Consensus achieved	Good agreement, with most ratings clustered around 4.	4.5	1.0	Consensus achieved	Good agreement, with most ratings clustered between 4 and 5.	4.0	1.0	Consensus achieved	Good agreement, with most ratings clustered around 4.
**Q13**	5.0	1.0	Consensus achieved	Good agreement, with most ratings clustered between 4 and 5.	5.0	1.0	Consensus achieved	Good agreement, with most ratings clustered between 4 and 5.	4.0	1.0	Consensus achieved	Good agreement, with most ratings clustered around 4.

Q1: No cabinets; Q2: No place for a break; Q3: Lack of teaching rooms; Q4: Too small teaching rooms; Q5: Too numerous student groups; Q6: Classes with the patient in a separate place; Q7: Too small rooms in clinics; Q8: Too many students to work with patients; Q9: Too few teaching activities; Q10: Lack of computer rooms; Q11: More self-learning methods; Q12: More Cased-Based Learning; Q13: More simulated classes. Me—median; IQR—interquartile range. Consensus was predefined as a median score ≥ 4.0 and an IQR ≤ 1.0. Items not meeting this criterion but demonstrating an IQR of 1.5 were classified as showing a high degree of agreement. Agreement categories were used to facilitate interpretation of panel responses. Agreement level classification based on IQR values: IQR ≤ 1.5 = high agreement; IQR = 2.0 = low agreement; IQR ≥ 2.5 = no agreement/no consensus. N/A—the indicator does not apply to a given subgroup of experts.

**Table 3:** Correlation matrix for responses to individual questionnaire items in the overall study group.

	Q1	Q2	Q3	Q4	Q5	Q6	Q7	Q8	Q9	Q10	Q11	Q12	Q13
**Q1**	-	**0.31**	0.05	0.16	**0.30**	0.13	**0.38**	0.18	0.03	0.09	0.03	−0.06	0.11
**Q2**	**0.31**	-	0.11	**0.32**	0.18	**0.51**	**0.46**	**0.32**	0.15	0.21	0.12	0.01	−0.04
**Q3**	0.05	0.11	-	**0.38**	0.14	0.08	0.13	**0.26**	0.17	0.09	−0.03	0.09	0.15
**Q4**	0.16	**0.32**	**0.38**	-	**0.39**	**0.28**	**0.45**	**0.33**	0.07	0.15	0.09	0.04	0.01
**Q5**	**0.30**	0.18	0.13	**0.39**	-	**0.23**	**0.40**	**0.32**	0.01	0.05	0.09	0.09	0.12
**Q6**	0.13	**0.51**	0.08	**0.28**	**0.23**	-	**0.29**	**0.21**	0.11	0.06	0.17	0.12	0.09
**Q7**	**0.38**	**0.46**	0.13	**0.45**	**0.40**	**0.29**	-	**0.31**	0.14	**0.28**	0.05	−0.02	0.08
**Q8**	0.18	**0.32**	**0.26**	**0.33**	**0.32**	**0.21**	**0.31**	-	0.01	0.12	0.08	**0.22**	0.08
**Q9**	0.03	0.15	0.17	0.07	0.01	0.11	0.14	0.01	-	**0.26**	0.06	0.03	0.08
**Q10**	0.09	0.21	0.09	0.15	0.05	0.06	**0.28**	0.12	**0.26**	-	0.09	−0.05	−0.12
**Q11**	0.03	0.12	−0.03	0.09	0.09	0.17	0.05	0.08	0.06	0.09	-	**0.32**	**0.29**
**Q12**	−0.06	0.01	0.09	0.04	0.09	0.12	−0.02	**0.22**	0.03	−0.05	**0.32**	-	**0.36**
**Q13**	0.11	−0.04	0.15	0.01	0.12	0.09	0.08	0.08	0.08	−0.12	**0.29**	**0.36**	-

Q1: No cabinets; Q2: No place for a break; Q3: Lack of teaching rooms; Q4: Too small teaching rooms; Q5: Too numerous student groups; Q6: Classes with the patient in a separate place; Q7: Too small rooms in clinics; Q8: Too many students to work with patients; Q9: Too few teaching activities; Q10: Lack of computer rooms; Q11: More self-learning methods; Q12: More Cased-Based Learning; Q13: More simulated classes. Bold values indicate statistically significant correlation coefficients when *p* < 0.05.

**Table 4:** Correlation matrix for responses to individual questionnaire items among students.

	Q1	Q2	Q3	Q4	Q5	Q6	Q7	Q8	Q9	Q10	Q11	Q12	Q13
**Q1**	-	**0.31**	0.05	0.16	**0.30**	0.13	**0.38**	0.18	0.03	0.10	0.03	−0.06	0.11
**Q2**	**0.31**	-	0.11	**0.32**	0.19	**0.51**	**0.46**	**0.32**	0.16	0.21	0.12	0.01	−0.04
**Q3**	0.05	0.11	-	**0.34**	0.02	0	0.10	0.10	0.16	0.15	−0.11	0	−0.80
**Q4**	0.16	**0.32**	**0.34**	-	**0.33**	**0.33**	**0.46**	**0.22**	0.17	**0.23**	0.14	−0.04	−0.11
**Q5**	**0.30**	0.19	0.02	**0.33**	-	0.20	**0.40**	**0.28**	0	0.18	0.10	0.04	0.05
**Q6**	0.13	**0.51**	0	**0.33**	0.20	-	**0.36**	**0.24**	0.05	0.13	0.20	−0.01	0
**Q7**	**0.38**	**0.46**	0.10	**0.46**	**0.40**	**0.36**	-	**0.32**	0.14	**0.28**	0.10	−0.08	0.03
**Q8**	0.18	**0.32**	0.10	**0.22**	**0.28**	**0.24**	**0.32**	-	−0.08	0.20	0.06	0.14	−0.8
**Q9**	0.03	0.16	0.16	0.17	0	0.05	0.14	−0.08	-	**0.29**	0.08	−0.12	0
**Q10**	0.10	0.21	0.15	**0.23**	0.18	0.13	**0.28**	0.20	**0.29**	-	0.11	0.03	−0.11
**Q11**	0.03	0.12	−0.11	0.14	0.10	0.20	0.10	0.06	0.08	0.11	-	**0.24**	**0.24**
**Q12**	−0.06	0.01	0	−0.04	0.04	−0.01	−0.08	0.14	−0.12	0.03	**0.24**	-	**0.27**
**Q13**	0.11	−0.04	0.80	−0.11	0.05	0	0.03	−0.08	0	−0.11	**0.24**	**0.27**	-

Q1: No cabinets; Q2: No place for a break; Q3: Lack of teaching rooms; Q4: Too small teaching rooms; Q5: Too numerous student groups; Q6: Classes with the patient in a separate place; Q7: Too small rooms in clinics; Q8: Too many students to work with patients; Q9: Too few teaching activities; Q10: Lack of computer rooms; Q11: More self-learning methods; Q12: More Cased-Based Learning; Q13: More simulated classes. Bold values indicate statistically significant correlation coefficients when *p* < 0.05.

**Table 5:** Correlation matrix for responses to individual questionnaire items among academic teachers.

	Q3	Q4	Q5	Q6	Q7	Q8	Q9	Q10	Q11	Q12	Q13
**Q3**	-	**0.36**	0.27	0.16	0.18	**0.43**	0.28	0.06	0.13	0.25	0.25
**Q4**	**0.36**	-	**0.46**	0.16	**0.44**	**0.44**	−0.05	0.09	0.04	0.13	0.24
**Q5**	0.27	**0.46**	-	0.26	**0.46**	**0.31**	0.12	−0.14	0.15	**0.34**	0.27
**Q6**	0.16	0.16	0.26	-	0.15	0.16	0.27	−0.12	0.13	**0.38**	0.26
**Q7**	0.18	**0.44**	**0.46**	0.15	-	**0.32**	0.15	0.30	−0.02	0.10	0.18
**Q8**	**0.43**	**0.44**	**0.31**	0.16	**0.32**	-	0.29	0.15	0.18	**0.31**	**0.40**
**Q9**	0.28	−0.05	0.12	0.27	0.15	0.29	-	0.15	**0.35**	**0.47**	0.30
**Q10**	0.06	0.09	−0.14	−0.12	0.30	0.15	0.15	-	0	−0.14	−0.10
**Q11**	0.13	0.04	0.15	0.13	−0.02	0.18	**0.35**	0	-	**0.55**	**0.42**
**Q12**	0.25	0.13	**0.34**	**0.38**	0.10	**0.31**	**0.47**	−0.14	**0.55**	-	**0.60**
**Q13**	0.25	0.24	0.27	0.26	0.18	**0.40**	0.30	−0.10	**0.42**	**0.60**	-

Q3: Lack of teaching rooms; Q4: Too small teaching rooms; Q5: Too numerous student groups; Q6: Classes with the patient in a separate place; Q7: Too small rooms in clinics; Q8: Too many students to work with patients; Q9: Too few teaching activities; Q10: Lack of computer rooms; Q11: More self-learning methods; Q12: More Cased-Based Learning; Q13: More simulated classes. Bold values indicate statistically significant correlation coefficients when *p* < 0.05.

## Data Availability

The original contributions presented in this study are included in the article. Further inquiries can be directed to the corresponding authors.

## References

[1] Cantillon P., Hutchinson L., Wood D. (2003). ABC of Learning and Teaching in Medicine.

[2] Burgess A., van Diggele C., Roberts C., Mellis C. (2020). Key tips for teaching in the clinical setting. BMC Med. Educ..

[3] Bedoll D., van Zanten M., McKinley D. (2021). Global trends in medical education accreditation. Hum. Resour. Health.

[4] Cumming A. (2010). The Bologna process, medical education and integrated learning. Med. Teach..

[5] Stryjski R., Stryjski A. (2016). Systemy kształcenia lekarzy w wybranych krajach europejskich (studia lekarskie jedno—Czy dwustopniowe?). Probl. Profesjologii.

[6] Sejm of the Republic of Poland Ustawa z Dnia 5 Grudnia 1996 r. o Zawodach Lekarza i Lekarza Dentysty [Act of 5 December 1996 on the Professions of Physician and Dentist]. Dz. U. 1997, No. 28, Item 152. https://isap.sejm.gov.pl/isap.nsf/DocDetails.xsp?id=WDU19970280152.

[7] Sejm of the Republic of Poland Ustawa z Dnia 28 Kwietnia 2011 r. o Zmianie Ustawy o Zawodach Lekarza i Lekarza Dentysty [Act of 28 April 2011 Amending the Act on the Professions of Physician and Dentist]. Dz. U. 2011, No. 113, Item 658. https://www.adwokatura.pl/admin/wgrane_pliki/file-ustawalekarzedentysci-29450.pdf.

[8] Ramani S., Leinster S. (2008). AMEE Guide no. 34: Teaching in the clinical environment. Med. Teach..

[9] Harden R.M., Crosby J.R. (2000). AMEE Guide No 20: The good teacher is more than a lecturer: The twelve roles of the teacher. Med. Teach..

[10] Prideaux D., Alexander H., Bower A., Dacre J., Haist S., Jolly B., Norcine J., Roberts T., Rothman A., Rowe R. (2000). Clinical teaching: Maintaining an educational role for doctors in the new health care environment. Med. Educ..

[11] Boyle M., McKenna L. (2016). Paramedic and midwifery student exposure to workplace violence during clinical placements in Australia—A pilot study. Int. J. Med. Educ..

[12] Beckman T.J., Lee M.C. (2009). Proposal for a collaborative approach to clinical teaching. Mayo Clin. Proc..

[13] Le K.D.R., Downie E., Azidis-Yates E., Shaw C. (2024). The Impact of Simulated Ward Rounds on the Clinical Education of Final-Year Medical Students: A Systematic Review. Int. Med. Educ..

[14] Martín-Parrilla M.Á., Durán-Gómez N., Nadal-Delgado M., López-Jurado C.F., Palomo-López P., Cáceres M.C. (2026). Implementing and Evaluating a Comprehensive Simulation-Based Program to Enhance Nursing Students’ Coping with Death, Burnout, and Communication Skills. Front. Psychol..

[15] Li F., Zhao H., Huang Q., Zhang C., Chen G. (2026). Development and validation of a competency-based evaluation framework for clinical medical trainees in the era of artificial intelligence: A mixed-methods study in China. BMC Med. Educ..

[16] Hays R., Hays R. (2006). Teaching and learning in clinical settings. Teaching and Learning in Clinical Settings.

[17] Aluko A., Rana J., Burgin S. (2018). Teaching & Learning Tips 8: Preparing to teach in ambulatory settings. Int. J. Dermatol..

[18] Daily J.A., Bolin E., Eble B.K. (2018). Teaching pediatric cardiology with meaning and sense. Congenit. Heart Dis..

[19] Huang P.H., Haywood M., O’Sullivan A., Shulruf B. (2019). A meta-analysis for comparing effective teaching in clinical education. Med. Teach..

[20] Smith J.R., Lane I.F. (2015). Making the Most of Five Minutes: The Clinical Teaching Moment. J. Vet. Med. Educ..

[21] Khodyakov D., Grant S., Kroger J., Bauman M. (2023). Document No. TL-A3082-1. RAND Methodological Guidance for Conducting and Critically Appraising Delphi Panels.

[22] Gattrell W.T., Logullo P., van Zuuren E.J., Price A., Hughes E.L., Blazey P., Winchester C.C., Tovey D., Goldman K., Hungin A.P. (2024). ACCORD (ACcurate COnsensus Reporting Document): A Reporting Guideline for Consensus Methods in Biomedicine Developed via a Modified Delphi. PLoS Med..

[23] Birko S., Dove E.S., Özdemir V. (2015). Evaluation of Nine Consensus Indices in Delphi Foresight Research and Their Dependency on Delphi Survey Characteristics: A Simulation Study and Debate on Delphi Design and Interpretation. PLoS ONE.

[24] Esteghamati A., Baradaran H., Monajemi A., Khankeh H.R., Geranmayeh M. (2016). Core components of clinical education: A qualitative study with attending physicians and their residents. J. Adv. Med. Educ. Prof..

[25] Nasa P., Jain R., Juneja D. (2021). Delphi methodology in healthcare research: How to decide its appropriateness. World J. Methodol..

[26] Hakim A. (2023). Investigating the challenges of clinical education from the viewpoint of nursing educators and students: A cross-sectional study. SAGE Open Med..

[27] Magnier K.M., Wang R., Dale V.H., Pead M.J. (2014). Challenges and responsibilities of clinical teachers in the workplace: An ethnographic approach. J. Vet. Med. Educ..

[28] Magnier K., Wang R., Dale V.H.M., Murphy R., Hammond R.A., Mossop L., Freeman S.L., Anderson C., Pead M.J. (2011). Enhancing clinical learning in the workplace: A qualitative study. Vet. Rec..

[29] Jafarian-Amiri S.R., Zabihi A., Qalehsari M.Q. (2020). The challenges of supporting nursing students in clinical education. J. Educ. Health Promot..

[30] Shirazi M., Akbari L., Babaee M. (2013). Assessment of the condition of clinical education from the viewpoints of undergraduate nursing students: Presentation of problem-oriented strategies. J. Nurs. Educ..

[31] Abazari P., Namnabati M. (2017). Restrictive and facilitating factors of nursing students clinical education effectiveness. Iran. J. Med. Educ..

[32] Flores-Sánchez A.R., Gutiérrez-Cirlos C., Sánchez-Mendiola M. (2022). Analysis of physical learning spaces in a university hospital: A case study. Med. Teach..

[33] Vakili R., Vakili S., Ajilian Abbasi M., Sh M., Saeidi M. (2024). Overcrowded Classrooms: Challenges, Consequences, and Collaborative Solutions for Educators: A Literature Review. Med. Educ. Bull..

[34] Shirani Bidabadi N., Nasr Isfahani A., Rouhollahi A., Khalili R. (2016). Effective Teaching Methods in Higher Education: Requirements and Barriers. J. Adv. Med. Educ. Prof..

[35] Stoumpos A.I., Stoumpou R.I. (2025). Modern Digital and Technological Educational Methods. Trends High. Educ..

[36] Patterson J.A., Chrisman M., Skarbek A., Martin-Stricklin S., Patel S.E. (2021). Challenges of Preparing Nursing Students for Practice: The Faculty Perspective. J. Nurs. Educ..

[37] Schifano J., Niederberger M. (2025). How Delphi studies in the health sciences find consensus: A scoping review. Syst. Rev..

